# Neurobiological limits and the somatic significance of love: Caregivers’ engagements with neuroscience in Scottish parenting programmes

**DOI:** 10.1177/0952695120945966

**Published:** 2020-10-21

**Authors:** Tineke Broer, Martyn Pickersgill, Sarah Cunningham-Burley

**Affiliations:** 151025 University of Edinburgh, UK; 151025 University of Edinburgh, UK; 151025 University of Edinburgh, UK

**Keywords:** expertise, neuroscience, parenting, sociology, subjectivity

## Abstract

While parents have long received guidance on how to raise children, a relatively new element of this involves explicit references to infant brain development, drawing on brain scans and neuroscientific knowledge. Sometimes called ‘brain-based parenting’, this has been criticised from within sociological and policy circles alike. However, the engagement of parents themselves with neuroscientific concepts is far less researched. Drawing on 22 interviews with parents/carers of children (mostly aged 0–7) living in Scotland, this article examines how they account for their (non-)use of concepts and understandings relating to neuroscience. Three normative tropes were salient: information about children’s processing speed, evidence about deprived Romanian orphans in the 1990s, and ideas relating to whether or not children should ‘self-settle’ when falling asleep. We interrogate how parents reflexively weigh and judge such understandings and ideas. In some cases, neuroscientific knowledge was enrolled by parents in ways that supported biologically reductionist models of childhood agency. This reductionism commonly had generative effects, enjoining new care practices and producing particular parent and infant subjectivities. Notably, parents do not uncritically adopt or accept (sometimes reductionist) neurobiological and/or psychological knowledge; rather, they reflect on whether and when it is applicable to and relevant for raising their children. Thus, our respondents draw on everyday epistemologies of parenting to negotiate brain-based understandings of infant development and behaviour, and invest meaning in these in ways that cannot be fully anticipated (or appreciated) within straightforward celebrations or critiques of the content of parenting programmes drawing on neuropsychological ideas.

## Introduction

While parents have since long received guidance on how to raise children, a relatively new element of this involves explicit references to the development of infant brains (Lowe, Lee, and Macvarish, 2015a, 2015b; Macvarish, Lee, and Lowe, 2014; Pickersgill, 2014). Emerging from the US in the early 1990s (Macvarish, Lee, and Lowe, 2014), these ideas about children’s brain development – also known as ‘brain-based parenting’ (Hughes and Baylin, 2012) – are related to, and exacerbated by, what some refer to as a culture of ‘intensive motherhood’ or ‘intensive parenting’. Sociological literature on this issue has argued that in such discourses on intensive parenting, parents (and particularly mothers) are seen as almost exclusively responsible for how their children grow up (see, for example, Bell, McNaughton, and Salmon, 2009; Lee, 2008; Lupton, 2011; Romagnoli and Wall, 2012; Thornton, 2011; Wall, 2010). These discourses are related to, and sometimes seen as a continuation of, so-called ‘attachment-based parenting’. This draws on psychological theories of maternal/infant attachment, and similarly argues for the importance of secure and loving parent–child relationships in determining the future of the child (Lee, Macvarish, and Lowe, 2014; Pickersgill, 2018). Scientised notions of parenting have circulated widely, with a range of UK policy documents discussing children’s (brain) development and often mentioning the adverse consequences of suboptimal parenting (Broer and Pickersgill, 2015b; Lowe, Lee, and Macvarish, 2015a, 2015b).

The assumptions and claims underlying ‘brain-based parenting’ are not uncontested, either within academic writing (Lowe, Lee, and Macvarish, 2015a, 2015b; Macvarish, Lee, and Lowe, 2014, 2015) or among policy and practice actors themselves (Broer and Pickersgill, 2015a, 2015b). Critics commonly assert that the cultural traction of neuroscience makes ideas around ‘brain-based parenting’ compelling, with non-scientists in particular deemed easily swayed by the iconography of brain scans (Weisberg *et al.*, 2008). As Lee, Macvarish, and Lowe (2014: 14) have reflected, family policies based on neuroscience constitute ‘an intensified focus on intimate interactions between the parent and child (such as feeding, touch, speaking)’. They argue that these interactions are regarded by governments as appropriate targets of intervention for an ever-larger proportion of the population.

Alongside arguments about the allure of neuroscience within policy and practice, criticisms also focus on the effects of parents’ use of brain-based ideas in the raising of their children. For instance, some have asserted that ideas about parenting based on neuroscience revolve around imposing middle-class values (and associated assumptions about educational, financial, and temporal resources) on working-class families. These include seeking to stimulate and maximise intellectual development and attainment as early as possible (Perrier, 2013; Romagnoli and Wall, 2012). Relatedly, Featherstone, Morris, and White (2014), for example, have argued that ideas about brain-based parenting increase the ability of social workers to remove children earlier and more frequently from families, should children be considered to be at risk. Where researchers have examined how parents engage with discourses of intensive or brain-based parenting, they often find that parents do indeed engage with these discourses to a large extent.^1^ Drawing on semi-structured interviews, Wall (2004, 2010) has shown how Canadian mothers try to adhere to enjoinders to optimise their children’s brain development, which results in parents becoming stressed and anxious about using their time ‘productively’. However, Romagnoli and Wall (2012) have also found resistance among parents to ideas associated with brain-based parenting. This is because such discourses are deemed by some to ask too much of parents in terms of the investment of time and energy, or because of a more fundamental disagreement over the extent to which brain-based parenting is actually beneficial for children.

In this article, we take cues from such work to investigate how parents negotiate understandings about children’s brains. Following authors such as Beaulieu (2003) and Derksen (2011), we argue that statements based on neuroscience can be considered generative; that is, they play a role in the constitution of parental affects, subjectivities, and care practices. Furthermore, to explore how ‘lay’ people respond to expertise/knowledge that refers to them or is (argued to be) relevant for them, we engage with the sociology of expertise. In doing so, we highlight how parents negotiate and sometimes resist expert guidance and knowledge claims. We base our analysis on semi-structured interviews with 22 parents/carers of (mostly) young children (generally aged from 0–7), all living in Scotland.

## Neuroscience and the self

While our analysis is a contemporary one, it speaks to a longer historical debate on how humans think about themselves, and the extent to and ways in which (neuro)sciences play a role in changing these understandings. This has, for instance, been discussed in a recent special issue on the future of the history of the human sciences. Smith (2019), in particular, makes an argument for allowing for multiple ways of understanding humanity, opposing biologically reductionistic accounts. We take seriously his call for more empirical studies into how people other than scientists engage with neurosciences, and whether they ‘understand…selfhood as brainhood and in what ways’ (ibid.: 12). Moreover, he argues that ‘the “history” in the history of the human sciences signals…a commitment *to keeping open* discussion about being human, drawing…on the debates that have gone on and continue to go on’ (ibid.: 16; emphasis in original). One way in which this discussion can be kept open is to examine how lay people understand themselves and their social and somatic worlds. It is in this spirit that we investigate how (parent and child) subjectivities come to be produced through particular (parenting) practices, and analyse what this means for wider understandings of being human.

The relationship between expert recommendations and parenting practices has been theorised and empirically examined extensively by historians (Mechling, 1975; Raftery, 1995; Wrigley, 1989). Both methodological and theoretical challenges have been documented and explored in relation to determining the extent to which recommendations from experts are a reflection of parenting practices, an encouragement to change these, or neither (Mechling, 1975). Given the long history of parenting recommendations and ideas, our analysis also lays further empirical specificity on how parents engage with scientific knowledge, and how parent and child subjectivities are produced through these engagements.

A particularly productive angle for historical studies of parenting ideas is in relation to theories of attachment. Raz (2014), for instance, has shown how two different theories of maternal deprivation (as relating to attachment) and sensory deprivation co-aligned in constructing what came to be seen as autism, and autism’s causes. Similarly, Harrington (2016: 96) traces the history of ‘mother love’ in relation to both healthy development of children now as well as ‘responsible citizenship’ down the line. She focuses on what has been referred to as ‘schizophrenogenic mother[s]’, who in their physical and emotional absence contributed to the emergence of schizophrenia in their children, and shows how from the 1960s and 1970s, mothers came to ‘speak…in their own increasingly politicized voices’ against this ‘mother blaming’, ‘invoking the alternative understanding of schizophrenia as a disease of bad biochemistry instead’ (ibid.: 111). Moreover, historians have shown how attachment has been appropriated to reinforce politically conservative parenting projects in which mothers are predominantly responsible for raising children, an individual responsibility going hand in hand with the notion that no extra resources from the state are needed (Duschinsky, Greco, and Solomon, 2015a). Of specific relevance is a study by Duschinsky, Greco, and Solomon (2015b) showing the specific historical and political routes of attachment theories into current policy reports on ‘Early Intervention’ (see, for instance, Allen, 2011; Allen and Smith, 2009), which draw on neuroscientific knowledge to consolidate attachment ideas.

Through this article, we aim to build on these historical and sociological literatures in order to offer an analysis of how parents today creatively and pragmatically use science, and also judge the information based on its do-ability, value-laden-ness, and historicity (with some research judged to be ‘out of date’ by our respondents). In what follows, we initially outline our theoretical inspirations, before describing our methods. Thereafter, our findings come in three subsections. First, we focus on a piece of brain-based information relating to children’s processing speed. Second, we turn to accounts of the so-called ‘Romanian orphans’: a collection of children who experienced extreme deprivation in Romanian state-run orphanages, with a range of neurodevelopmental consequences (Nelson, Fox, and Zeanah, 2014). Third, we document discussions around whether children should be left to cry, especially when they are going to sleep. In the discussion, we reflect on our analysis, and in particular on how parents negotiate brain-based ideas and understandings, and how children’s agency is constituted with and through these negotiations. Our analysis, then, is relevant to explorations of how notions about parenting, and in particular the contested ideas associated with brain-based parenting, are received, adapted, and rejected by parents themselves, and how parent and infant subjectivities are produced in these processes. Hence, we speak to a longer historical debate about how scientific notions are incorporated in ‘lay’ understandings and practices.

## Social studies of science, knowledge, and expertise

A key strand of literature that we draw on is work in the social studies of neuroscience. In particular, we take inspiration from research demonstrating the productive nature of brain images and the ways through which neuroscientific theories influence contemporary ideas of the self. Historians and sociologists have argued that the brain has become increasingly important to how people understand themselves, with neologisms like ‘brainhood’ (Vidal, 2009) and ‘neurochemical selves’ (Rose, 2007) invoked in such considerations. The importance of neuroscience is often linked to, but also contrasted with, knowledge from the psy-sciences, for example regarding the construction of categories like ‘depression’ (Rose and Abi-Rached, 2013). While neuroscience is often criticised for being reductionist, Beaulieu (2003: 563) has argued that it can be analytically more useful to consider investigations of the brain as semiotically generative: she encourages sensitivity to how neuroscientific work ‘powerfully redefines concepts like behaviour, nurture, culture and environment’. Gardner *et al.* (2018: 191) make a similar argument when they suggest the importance of investigating ‘how [neurological] potentials become culturally relevant’.

Work on the productive nature of neuroscience has some resonances with the scholarship of philosopher Ian Hacking, who has written extensively about how people respond to new categorisations and framings of subjects and their experiences (Hacking, 1999, 2007). In particular, we can see similarities in relation to the productivity of scientific knowledge for how people think about themselves and their social lives (for instance, in terms of the good, ‘attached’ mother, or the neurologically impoverished neglected infant). Moreover, both Hacking and work on the productive nature of neuroscience are indebted to Foucault’s thinking about the iterative relationship between knowledge and subjectivity (Foucault, 1977, 1978). Here, we share such scholars’ interest in, as Derksen (2011: 842) puts it, how ‘neuroscientific theories and classifications are sources of identity that are not only adopted, but also adapted and sometimes resisted by the people they apply to’.

We want to make links between this body of research and other research in the sociology of expertise. This emphasises how ‘lay’ people respond to expertise/knowledge that refers to them or is relevant for them. We find especially instructive work that shows how individuals are reflexive agents who do not necessarily passively accept scientific knowledge, but instead often critically reflect on, adjust, or reject expert knowledge. Sociologist Brian Wynne’s (1992) research is commonly judged path-breaking in this regard; he detailed how and why sheep farmers in Cumbria in the UK rejected knowledge about radioactivity (following an incident at a nearby nuclear reactor) based on local epistemologies of practice. In the realm of health and medicine, analysts of biomedicine and society have illustrated how responses to ostensibly novel technoscience are assessed against experiential knowledge (see, for example, Cunningham-Burley, 2006; Kerr and Cunningham-Burley, 2000; Kerr, Cunningham-Burley, and Amos, 1998; Kerr, Cunningham-Burley, and Tutton, 2007a, 2007b; Pickersgill, Martin, and Cunningham-Burley, 2015). Such work has illustrated how understandings of the body authorised within some academic settings can be rejected by other kinds of experts when they fail to align with pre-existing ontologies and epistemologies.

Sociologists and other social scientists have further studied how family actors negotiate parenting-related knowledge and expertise, with breastfeeding perhaps the most studied example. As a range of analysts have indicated (see Knaak, 2010; Ryan, Bissell, and Alexander, 2010; Wall, 2001), a strong moral discourse on ‘breast is best’ is publicly available within many nations; yet this is also sometimes contested by individuals targeted by it. Relatedly, what and how much children eat has been the subject of a notable and ongoing public debate, with sociologists in turn exploring these and resonant issues through accounts of parents’ responses to expert ideas about their children’s diet (see Bell, McNaughton, and Salmon, 2009; Keenan and Stapleton, 2010; Lupton, 2011; O’Key and Hugh-Jones, 2010). Murphy (2003: 433), for instance, studied mothers’ perspectives on child nutrition recommendations, elucidating various ‘rhetorical strategies’ used by women ‘to defend themselves against the charges of maternal irresponsibility that arise when their practices do not conform to expert medical recommendations’. Work in this vein is important to our analysis, since it sensitises us to the diverse responses that parents can have to ideas around brain-based parenting – which may challenge assumptions about how these might be adopted or otherwise engaged with.

Indeed, previous studies have indicated that different actors engage with neuroscientific notions in diverse ways (Pickersgill, Cunningham-Burley, and Martin, 2011; Singh, 2013). Based on such studies, Gardner *et al.* (2018: 191) have suggested that it might be productive to speak of ‘multiple neurosocialities’, where the ‘transformative effects [of neuroscientific developments] depend on how they become immersed in local practices by creative and pragmatic agents’ (ibid.: 190). Such literature, then, implicitly or explicitly connects ideas from the sociology of expertise with the sociology of neuroscience, discussing the multiple strands constituting subjectivities, and, like Beaulieu’s and Derksen’s work mentioned above, arguing against the presence of solely reductionistic models of neuroscience and practices of neuroscientists.

We build on this work by focusing on a case study in a parenting context, which, except for parents of children with certain neurologic conditions (Borgelt *et al.*, 2014; Singh, 2005; Whiteley *et al.*, 2017), has not frequently been the focus of studies in the sociology of the neurosciences. Focusing on three specific ideas about the brain and attachment that our respondents introduced in the interviews, we examine how a range of neuropsychological notions were accounted for as supporting or generating certain parenting practices, and entailed the construction of particular kinds of infant capacities and subjectivities. Taking cues from the sociology of expertise, we show how parents reflexively weigh and judge understandings and guidance. These adjudications underscore the role of everyday epistemologies of parenting in terms of how the somatic salience of love and care, as well as the neurobiological limits of agency, are constructed and understood.

## Methods

The research underpinning this articled was part of a Leverhulme Trust–funded project on ‘Neuroscience and Family Life: The Brain in Policy and Everyday Practice’ (funded 2013–15). A key study aim was to better understand how neuroscientific notions and findings shape policy regarding, social services for, and personal experiences of parenting. Among the data collected for the research were 22 semi-structured interviews with parents and caregivers of young children in Scotland. Interviews covered respondents’ perspectives on raising their children, the information and suggestions they received, the role of parenting programmes in disseminating these, and the place of the brain within such ideas on parenting.

Interviewees were recruited through two parent programmes (explained in more detail below), chosen as recruitment sites for pragmatic and theoretical reasons. Pragmatically, we had previously conducted interviews with people involved in developing and delivering these courses (see Broer and Pickersgill, 2015a), which helped to ensure access. Theoretically, we knew neuroscience was discussed in these courses, and therefore that most of the respondents we would recruited would, in principle, be able to talk about the import of understandings and ideas grounded in neuroscientific concepts and findings for their parenting practices.

One of the aims of both of these programmes was to provide parents with confidence, knowledge, and skills to bring up their children. Groups of parents (primarily mothers, though not exclusively) participated in the programmes; within them, each session consisted of a mixture of talks, videos, and discussion among parents. One of the programmes was delivered through primary schools as well as parenting support centres; the other was convened by a UK children’s charity and located in parent or community support centres. This diversity of locations also accounts for the relatively wide age range of the participants’ children; some of the children were at the end of primary school, whereas others were young babies. Parents encountered the former course through a range of pathways; for instance, some participated via their child’s school, while others took part as a consequence of their wider engagement with a community service for fathers, families, or another group. Both programmes drew on neuroscience and theories of attachment to enhance parents’ knowledge about infant development, although the former did so somewhat more explicitly (and was the programme from which the majority of our participants were recruited). Broer visited some of the centres where the courses took place in order to make initial contact with parents, and to better comprehend the contexts in which the programmes and respondents were situated.

The programmes targeted a range of people (including young mothers, fathers, and parents with generally slightly older children and living in more affluent areas), and our sample deliberately reflected this diversity. It included people from various backgrounds who brought their children up in wide-ranging circumstances, such as with or without social work involvement, with or without the concern that their children might be removed by the state, and with or without a partner. We asked our respondents if they would like someone else (such as their own parents or another person closely involved in the care of their child) to join them for the interview, in order to generate richer dialogue. Interviews were generally conducted with a programme participant and, depending on their situation and preferences, a grandparent of their child, their partner, a friend of the family, or a support worker. Ten of the interviews included at least two participants, often resulting in a paired interview format; 12 were conducted with a single participant. This, we suggest, reflected the challenges and isolation of some of the participants, in terms of social support. Three of the principal participants were fathers, whereas in three other interviews the father was present as a second respondent. One interview was conducted not with a parent, but with a main carer (specifically, the grandfather of the child, of whom he had custody). In every other case, the principal participant was a mother. All but two of the interviews took place at the home of the principal participant; the remainder were conducted in local centres where the parenting programme had taken place. Interviews usually lasted a little over an hour and were all conducted by Broer.

A first detailed coding of the interviews was undertaken, after which codes were grouped in the light of the questions we wished to interrogate for this paper; that is, what pieces of neuroscience-based information resonated (or not) with parents, and how these were spoken about. Three relevant topics were introduced by respondents in a large number of the interviews (17 in total): children’s neuropsychological processing speed (9 interviews); neglected Romanian orphans (10 interviews); and what to do with a sleepy, crying infant (7 interviews). While some participants mentioned all three topics, in five of the interviews none of them was (explicitly) discussed, although in two of these neuroscience was mentioned more generally. In the next sections, we present and reflect upon data regarding each of these tropes.

## Results

### The significance of speed

In this section, we examine how our study participants described learning about children’s ‘processing speed’; that is, the time it takes between a stimulus and a child’s response to it. To give a brief introduction to how this notion is discussed in child development literature, psychologist Robert Kail (2000: 52) describes in one review article that on ‘a wide range of motor, perceptual, and cognitive tasks in which participants must respond rapidly, a common pattern of age differences emerges: 8- to 10-year-olds typically respond at a speed that is 5 to 6 standard deviation units below the average speed for young adults’. Even adolescents, Kail argues, respond on average more slowly than adults. Here, we analyse how parents made sense of information about processing speed they encountered in the parenting programmes attended, including how the agency of children was (re)positioned in and through our respondents’ engagements with this.

The notion of processing speed was often mentioned by our respondents when the parenting programme was initially introduced within the interview, or following a prompt about the developing brain, such as in the following extract from an interview with a father (Interview 18):

R: But I find that, that [programme leader], and that [organisation] have been absolutely amazing and really helped me understand all of this, the side of child development and that and how the brain works and everything like that; how a child’s brain works and that.…It’s like telling your child you know something, it takes double the time for them to process what you’ve asked them to do than it actually does for a normal adult to process it. So.…

I: Double the time?

R: Yeah. So like for example, telling them to put their shoes on, you have to wait a few minutes for them to be able to understand it and for their brain to realise, ‘right, my mum or my dad – or somebody – has asked me to do this, this is what I need to do, this is how I’m going to do it, kind of thing;’ and for the brain to then tell them how to do it. Whereas you’re normally within five minutes, or a couple of minutes, going, [change in tone] ‘I’ve already told you to put your shoes on’.

I: Yes.

R: So you’re kind of having to remember that it’s because actually, it’s not because they’re being disobedient or anything like that, or that they’re, you know, not being helpful and that, it’s the fact that they actually maybe haven’t completely processed your request.

Similarly, the extract below comes from an interview with a mother (Interview 19) who felt that information about children’s processing speed, and neurological development more generally, enjoined her to recalibrate her parenting practices:

I: I heard that some of the courses also talk about children’s brain development.

R: Yes, fascinating!

I: Was it?

R: Because I can’t remember all of it now and I’ll have to read over it again. Like I say, some of it I knew, but some of the stuff they’ve discovered over the last sort of 20 years was *amazing* about the way that they [children] develop and stuff. And it just really helps you understand where they’re coming from a lot more. You know you think okay, that’s why they do that! That’s why they can’t get the gist of that, because they’re just not at the stage where they can do that yet. Something about how their…they can take seven times longer than an adult to respond, to process and respond to something that you’ve said to them; that helped a lot. That stuck with me.

I: How did that help you?

R: Because you realise when you tell them to do something or you’re trying to get their attention, especially when it’s time sensitive, like mornings, a stressy time of the day, and they don’t respond. You don’t immediately go: argh! And say it again. I said this! And get annoyed with them. Because they’ve just maybe not kind of processed it yet. They might actually still be figuring out what you said and be ready to act or respond to you.

This participant added that ‘the more you find out about the brain the more astounded you are at the way it actually functions’, and asserted that this new knowledge had helped her to become more patient with and understanding of her children. As is apparent from the preceding extract, ideas about neurological limits to children’s processing speed can serve to excise conflict from domestic interactions between parents and their children, through reducing the agency parents regard children as expressing. Many of the comments made by the mother in Interview 19 resonated with perspectives expressed by other parents/carers, with this extract in particular illuminating how information about the brain can be argued to act as a resource for impelling shifts in comprehending children’s actions. However, the respondent above also appreciated knowledge about things other than the brain: she argued that regardless of whether behaviour was underpinned by ‘something physical or mental or emotional or what have you’, it was a good thing ‘to have that [behaviour] explained’ – with such explanations contributing to enhanced understandings of her children. Hence, neuroscientific explanations for her played an important role – but not necessarily the most important – in understanding and interacting with her children.

Eight other parents likewise spoke about how, thanks to the parenting programmes, they now knew that their children were not misbehaving or merely ignoring the adults in their lives, but rather that it took longer for them to process information. One woman (Interview 12) noted that such knowledge encouraged her to communicate differently with her children:

Even if it sounds like you’re being a bit abrupt, it’s better just to give one task or ask them to do one thing at a time, rather than overloading them, because it takes them longer to process things. Seven seconds, I think they said, seven times longer, it takes their brain seven times longer to understand what you’ve asked them.…So just be patient, don’t ask them again. Just count to ten…give them time to understand it.

One parent (Interview 4), however, reflected that while notions of processing speed enhanced her understanding of why children did not immediately respond to requests, frustration nevertheless remained:

[The parenting programme] taught you about, just like why, like it would maybe tell you why kids seem to be ignoring you when you’re speaking to them. Just that basically that their brain is not…all the different tracks of their brain haven’t developed so it takes something like seven times longer for a child to understand an order than it would an adult. So, if you tell them to get their shoes on it would take a lot longer than if you told an adult. An adult would click straight away and know exactly what you’re trying to tell them. Things like that, that definitely made a huge difference, ‘cause it is quite frustrating sometimes when you’re repeating yourself and it does feel that you’re being ignored. Obviously knowing things like that, you know that it’s not just your kid, that if they’re not understanding straight away.…Most of the time I’ve got more patience for [son] knowing that he is actually just trying to process what I’m saying rather than just ignoring me. Not all the time. It’s still frustrating whether you know why they’re doing it or not.

Similar to the comments above, two mothers who took part together in the interview (Interview 3) and who had both participated in a parenting programme said that even though children’s brains might take longer to process information, this does not mean that children never ignore their elders. Consequently, parents need to take suggestions about parenting ‘with a pinch of salt’:

R1: You’ll be like, ‘he’s bloody ignoring me’. But actually, they were nae [not] ignoring you. They were just…it was processing it and every time you said it again, they were processing it again, so it was taking them so much longer. So things like that.…But it’s all very good on paper and it’s all very good to teach people this, but.…

R2: It’s totally different in life.

R1: …it’s totally different in life.…‘Cause it says that, ‘oh when a child does this, like, it’s not that they’re ignoring you’. And that’s fair enough, but there is going to be once in that day when that child’s going to ignore you, so you can’t put it always down to the fact that they’re just taking longer to process it. There is at least five times a day that [son] will ignore me. And if I put it down to just the fact that, ‘oh he’s just taking longer to process it’, he’d be walking all over me. Do you know what I mean, so it’s, like, taking everything they say which is good and it’s great and it’s nice to be able to understand it, but it’s taking it all with a pinch of salt…like, you need to decipher what’s…you need to pick your battles really as well, like, kind of thing.

Hence, these respondents did not question the veracity of the neuroscientific claims about processing speed, nor its relevance as such – but they did render problematic its universal applicability. In effect, respondents drew upon epistemologies of everyday parenting to suggest that judgements necessarily had to be made about childhood agency, in order to ascertain whether or not one’s offspring were ‘really’ ignoring the requests of the adults in their lives.

In this section, we have seen how knowledge about neurological function and development, and about how children’s brains differ from those of adults, is accounted for by parents as a resource that encourages them to feel more patient with their children, and in some ways to expect less of them. Ideas about processing speed frame interactional conflict as an epiphenomenon of a biological limitation, reconstructing children as less intentional and ill-behaved in the process. While evidently (biologically) reductive, such processes of neurologisation in the accounts of parents are also generative (see Beaulieu, 2003) in terms of how they propel new understandings of childhood development, stimulate empathy regarding life course–related capacities, and shift parental practices in response to novel knowledge. Moreover, reductionism can be selective and partial, as parents exercise their own agency in applying neuroscientific ideas to their children in ways that still leave considerable room for autonomy: children, it is argued, sometimes *do* ignore their parents, regardless of the time taken to process the requests of their caregivers. As a result, parents still have to decide when to reprimand, get annoyed, or be patient; neuroscience does not (completely) solve the problem of agency and disobedience.

### The multiple lives of the Romanian orphans

The second brain-related story many respondents mentioned was associated with the so-called ‘Romanian orphans’. After Nicolae Ceauşescu was overthrown in Romania in 1989, it was discovered that ‘as many as 170,000 children’ were raised in state-run institutions, and were often ‘poorly cared for’ (Nelson, Fox, and Zeanah, 2014: 12). The orphans had not generally experienced the same exposure to sensorial stimuli as Romanian (and many other) children typically did, and adults did not commonly interact with them, or attend to even their most basic needs (ibid.: 2). A CT scan of the ‘extremely neglected’ brains of the orphans – produced by researchers from the US Child Trauma Academy in 2002 (Perry, 2002) – has come to circulate widely. For instance, a reproduction of the image featured on the title page of two key UK documents relating to early years policy (Allen, 2011; Allen and Smith, 2009), juxtaposed with an image of a ‘normal’ brain. As a result, narratives built around the Romanian orphans have contributed to the establishment of early intervention programmes across the UK (Broer and Pickersgill, 2015a, 2015b; Lee, Macvarish, and Lowe, 2014).

In 10 of the interviews, respondents mentioned the ‘true story’, as one father (Interview 18) referred to it, of the Romanian orphans. As we saw above, when telling tales of processing speed, parents talked largely about how children processed information seven times more slowly than adults, and used the example of putting on shoes, and getting ready to leave the house more generally, to illustrate childhood processing lag. The story of the Romanian orphans, though, was remembered in sometimes strikingly different ways, and the lives of the orphans took on diverse fates across the interviews. Indeed, one participant asserted to her friend that the children all died. Furthermore, while all the respondents who brought up processing speed unanimously described this knowledge as helpful, the relevance of neglected Romanian orphans to their lives was far more contested.

Some of the respondents who mentioned the Romanian orphans spoke of their growth being ‘stunted’ as a result of a lack of interaction. Such talk extended the somatic significance of love (including constitutive practices of care and attention) outwards from the brain to the body as a whole, as in the following extract from an interview with a mother (Interview 4):

Aye, they were never loved, they were never held or anything, and they never grew in size, they never aged. Just like strange developmental things that you think just come no matter what happens to you. But, yes they stayed small forever.…I think it showed you some that were rescued and they were like 25 year old and they looked about five year old, like never really grew or developed in any sort of way. Never learnt to speak obviously or feed themselves, things like that. Obviously that’s an extreme case, but it just shows you like if you’re not cuddling your child and nurturing your child and loving them, that it effects loads of different aspects, rather than just their behaviour or whatever.

As another participant (Interview 11), who had recently trained to become a programme facilitator, put it:

R: Yeah, sleep is important. Play is important, touch is important. Touch, if you don’t touch your child, and you don’t cuddle your child, and interact with your child, it can stunt their growth.

I: Oh really?

R: There was a picture, which is quite haunting, and it was in a Romanian village, in a Romanian orphanage. And there was children sitting at a table, and they looked about three and four year old – they were 18, 19 year old. And they were just so tiny, it was.…But it’s just, it’s a shocking image. You say, like throughout the whole session, it’s like how important it is to physically touch and hold your child. Which is why breastfeeding is just so publicised, it’s like, please just do it for the good of your child, and bonding wise. It’s, it’s really haunting to see, in a group, and you say, how old do you reckon they are. And there’s obviously a big boy there, so he’s a teenager, he’s maybe 20 years old, or something. And he’s got a little person on his knee, and he’s sort of looking down, and that little person that’s on his knee is, like, 18 years old.

I: Oh really?

R: And they can’t walk, ‘cause they’ve just been left, they can’t talk, they can’t do anything. And again, it’s about their brain development, how the fight or flight affects them, and stuff like that.

The final interview (Interview 22) we conducted was with a mother (R1) and father (R2) of one child. The female participant had completed the parenting programme; both respondents spoke of the emotive nature of some of the information it had offered:

I: So what kinds of things were emotional?…

R2: Didn’t you say there was some, was it a video, of some very deprived children or quite older children or adults who looked like.…

R1: It was a still, it was a photo of, yeah, children, and the course leader had kind of said how old do you think the children are? And I think most of us said five to maybe one of the oldest ones was maybe eight, nine I think was the.…And it turned out they were something like 15 to 20 or something. It was something horrendous.…I mean they were just physically…even if you thought you were maybe a year or two out you were.…This was really quite shocking. I think it was a Romanian orphanage or something, some, some really quite extreme kind of setting.

But I think that was a shock at just how bad it could be. But I think although that was very distressing I think for me what was quite upsetting [teary voice] was that the woman…she worked in the social work department, and she said, she made the point of saying don’t dismiss that as being Romanian orphanage focused, that there, I think the stat she gave was there’s one in three children in [city] right now who may not fall into as quite an extreme category, but who would definitely be physically and emotionally deprived like that. And I guess it’s that…I find that quite distressing. [teary] Just yeah, how unfair by luck of birth or misfortunate of birth that yeah, those kinds of differences.…

This mother said that hearing about the orphans did not result in changes to how she parented her son, not least because the course tutor asserted that she could ‘pretty much guarantee that none of you in this room, none of your kids will be affected by this directly’. However, the mother reflected that hearing about the Romanian orphans might make her somewhat more understanding towards a ‘naughty child at high school or the rough child’. She argued that schools, for instance, should not punish a bully but instead support them, ‘because they can’t really be in a good place if they’re behaving that way’.

Most respondents said that the story of children in Romanian state orphanages did not stimulate different parenting practices, since they gave their children love and affection anyway. A grandfather recounted how he sang to his granddaughter (for whom he was a key carer) on the bus, but that when he was a father he would have felt too ‘embarrassed’ to do so (Interview 13). Now, though, he could be ‘silly’ as that was what grandparents were meant to be. When asked if he talked and sang more to the child after hearing about the Romanian orphans and about the child brain absorbing information, he said:

I think it’s probably more with that because it gave me an awareness that when I’m speaking to her, her brain is absorbing. I would say, it made me.…Although I still spoke to her it made me more aware that what I was saying meant something to her and she could store it and use it so, aye, I would say that definitely, but beforehand I would just tell her stories and all the rest of it and not think anything of it but now when I’m telling her stories and things like that I’m aware that it’s maybe making a difference and it is going in her head. That sort of thing.

On a minority of occasions, however, interviewees indicated changes they had made after the programme. For instance, a father (Interview 18) narrated the story of the Romanian orphans and argued that it should be taught to ‘school age’ young people in order to prepare them for having children and the responsibilities it entails. He asserted that this cautionary tale would have been helpful for him had he heard it before becoming a parent, because ‘when [oldest son] was born, I knew nothing about how to look after a child or anything like that at all’. The respondent had participated in a parenting programme following the birth of his second child, and spoke about differences in how he interacted with his oldest son versus his youngest. This divergence was accounted for as relating to the information he had received through the programme:

Because I knew how important it is, so I would have done a lot more. So I’m now doing that with [youngest son], I know, now that I know how important it is I’m doing a lot more with [youngest son]; I’m singing a lot with him, and talking with him all the time and that. Even when I’m on the bus, just sitting on the bus, on a bus journey, I would never have one minute with silence, I will always be communicating with [youngest son], or [oldest son] now; whereas when [oldest son] was a child there maybe would have been times where maybe ten, 15, 20 minutes would go past without any communication, you know. But now that I know how important it is and everything like that, it is quite crucial.

Other participants were more critical about the place of Romanian orphans within parenting programmes. One mother (Interview 20), for instance, described how much of the content of the programme was ‘common sense’; when asked for an example, she replied:

They were talking about emotional well-being and the importance of that. They talked about, you know, development of the brain.…They wouldn’t go into loads of, sort of, you know, the anatomy or anything like that, it was just talking about development, brain importance, importance of having a bond with the babies, showing love, you know, things that we all do. They talked a lot about, not a lot, they talked about children who are neglected. They talked about orphans in Romania or when the brain doesn’t develop because babies are neglected and that wasn’t applicable to any of us there, you know. We were all caring mums and I thought.…So there was that, you know, the brain doesn’t develop as well. It was just more, hm, I can’t really remember, but there was nothing that made me think, ‘oh, wow, wow, that’s new’ and it was, kind of, like just reinforcing what we maybe already knew.

Another woman (Interview 9) was a critic of the parenting programme more broadly, and suggested that it should be tailored to infant age and socio-economic circumstance. She explained how the Romanian orphans featured in the programme, and referred to this as ‘out of date’. The respondent went on to describe her discomfort upon hearing of the deprivation the children had experienced, as well as her unease regarding the effects of this knowledge on the tenor of the programme:

R: I am very sad that there are children like that in this world. I wish there wasn’t, but I just wasn’t entirely.…I don’t know, it was a bit kind of.…I mean it was quite an old photograph and I just.…I don’t know, it made me feel a bit uncomfortable actually.

I: Why did it make you feel uncomfortable?

R: Well. Well, I didn’t want to say it, but I didn’t think it was actually relevant to the course particularly. It was a bit too extreme. And then we had this whole discussion about well, ‘isn’t it awful’, blah-blah-blah. And I just thought this isn’t why we’re here.

As she stated with humorous irony, this mother found the guidance relating to the Romanian orphans and loving your child ‘very obvious, so obvious that even we were managing to do it’. However, she argued that tales of the Romanian orphans and related notions of interacting more with children might have been more useful for parents in other socio-economic circumstances, who ‘were brought up differently’ and who had ‘no example set for you, so you don’t know’ about the import of overt affection. Thus, even though many parents did not talk about how information about neglected Romanian infants directly impacted their own parenting practices, the narrative served as a resource through which to craft dichotomous, class-based accounts of good versus bad (or ignorant) parenting.

In this section, we have seen that some respondents saw the ‘extreme’ (Interviews 4, 9, and 22) case of infants in state-run Romanian orphanages as having relevance for better understanding children and their needs. Yet other parents argued that it was unnecessary (and perhaps even offensive) to narrate tales of now adult orphans from post-dictatorship Romania within Scottish parenting programmes attended by ‘caring mums’. Though the veracity of the information presented was not (generally) critiqued, its relevance to the domestic lives of the respondents was reflexively judged and weighted. Many asserted that the information did not impact their parenting practices, arguing that they gave their children love and cuddles anyway. However, even they sometimes concluded that tales of the Romanian orphans promoted better understanding of children’s emotional and physical needs, and perhaps especially of *other* people’s offspring. Furthermore, in one instance, other adults were suggested to be appropriate beneficiaries of knowledge about affection and interaction, and in another case, a mother recounted the course tutor as suggesting this. In the process of presenting knowledge about the Romanian orphans as relevant for others, then, dichotomies were introduced or reinforced between ‘caring mums’ and others who (they considered) might not know better because of their own childhood experiences.

### Distress and development

In this final results section, we address ideas on infant sleep proffered in parenting programmes, and in particular on whether or not to leave a baby to cry. In the literature and elsewhere, this is widely referred to as infant ‘self-settling’ (Middlemiss, Yaure, and Huey, 2015), with behavioural interventions in which a crying child is gradually left alone for longer periods before being attended to described as ‘graduated extinction’ or ‘controlled crying’ (Honaker and Meltzer, 2014). Some researchers have argued that this method is effective for reducing infant sleep problems; however, they also consider that it can be emotionally challenging for parents (ibid.). Some developmental studies, drawing on theories of attachment and elements of the neurosciences, assert that babies’ levels of cortisol (popularly known as a ‘stress hormone’) increase when distress is not responded to (Philbrook and Teti, 2016).

In our research, some respondents discussed ideas about self-settling in relation to the Romanian orphans discussed above, but generally it was treated separately. As one woman (Interview 11) reflected:

There was a study done on children, to see when they were babies, you know like, how some of them say leave them, leave them to cry themselves to sleep. That’s how it’s supposed to be, and once they’re fed, and they’re changed, and they’re comfy, leave them. Another one says, no you cuddle them, you soothe them, and you talk to them. The fight or flight [response] in a child that’s been left is higher than the child that’s been soothed. Because they know nothing else apart from crying, and crying doesn’t work, so they eventually stop crying, which is quite sad.

Infant sleep was sometimes mentioned within more general talk around the importance of demonstrably loving one’s child. One mother talked about giving her children love and attention, saying ‘you can’t spoil a child with too much love’ (Interview 20). She contrasted affect-laden interaction with what she described as ‘plonking’ a baby in front of the television, asserting that a parent is ‘the best toy’ for babies in terms of stimulating brain development. She went on as follows:

R: I think that’s important and that’s what the course taught me, as [well as being] a midwife, the importance of that bonding, you know, controlled crying not necessarily is a good thing, you know.

I: What is controlled crying?

R: Controlled crying is when you leave your baby to cry, you know, just letting it learn to settle itself down by itself.

I: I see.

R: The brain is just so not developed and has to learn to feel safe and sometimes by feeling safe, that’s how you can comfort yourself, not by crying yourself to sleep.

In one interview (Interview 10), a respondent stated that ‘distress can destroy neural pathways’, before correcting herself to instead note that distress was not ‘positive for the development of a child’s brain’. This woman had slightly older children, and said that when her eldest was a baby the guidance she encountered was to leave a baby to cry rather than to co-sleep (that is, to sleep in the same bed as a young infant). Today, though, an emphasis on infant attachment might result in quite different enjoinders:

R1: The course talks about attachment theories, which I thought was really interesting, and looking back now I think we would have probably been more helpful to bring up my eldest in a much stronger attachment theory style of parenting rather than expecting him you know to settle himself and he’s in his room, we’re in our room, that kind of quite old.…I would look back now and think God, that’s actually quite old fashioned.

I: So you would have done it differently.

R1: Yeah. I’d have had him in my bed, I’d have co-slept with him. I’d have, you know.…Yeah, if I could turn the clock back, yeah, I’d do certain things differently. But I wouldn’t, I got all the other stuff, I’m very happy with all the other stuff that I did, all the interactions, and I spent loads of time with him, and that’s perfect, and that was lovely memories.

We can see, then, that notions of attachment and neurological development can provide sources of knowledge for parents to reframe past parenting practices as imperfect, and potentially deterministic in leading to certain outcomes. At the same time, however, the inability to change choices made months or years ago can serve to dilute the salience of such knowledge: while the mother in Interview 10 described regret for not having co-slept with her eldest son, she also asserted that co-sleeping would not have had a significant impact on his personality:

But, yeah, just that little bit about his life, around sleep and settling, and I realise now that he was probably, he *was* quite an anxious young child. We didn’t pick up his anxiety till a bit later on in his life, and he does have anxiety. So that was interesting, but I haven’t given myself a hard time about a lot of this, that perhaps if we’d have parented him differently he wouldn’t have, he wouldn’t have had anxiety. But I don’t think that would have made a huge difference.

Some parents spoke of how they listened to and heeded advice more carefully and dutifully with their first child, but that they became more confident in their own expertise with later children. Others described how the propensities of their children rendered problematic the advice they had received. In the following extract (Interview 16), one woman (R1, who had a young daughter) and her own mother (R2, who was heavily involved in raising her granddaughter) talked about receiving advice that did not lead to the changes in R1’s daughter that it was aimed at achieving:

R2: Sometimes the biggest teacher’s the child as well.

R1: Yeah. So…it is. With [daughter], you can have all this different advice from health visitors, [parenting support organisation], even.…They mostly are all the same advice, but you could have all the advice in the world, but the only person that’s going to really teach you is [daughter]. Because what their facts can tell you could be completely different for [daughter].

I: Can you give an example of that?

R1: …[Daughter] used to be really bad for going to sleep. It’s not that she was bad, but if she didn’t want to go to sleep, she would not go to sleep. And [family nurse] said to me, ‘right, you’re just going to have to ride her out. Let her cry. Let her scream. Let her tantrum. Just leave her. As long as you’re going in and checking her to make sure that she’s actually…she’s not crying because she’s hurt herself, she’s crying because she’s in a tantrum’. Now I rode it out for two and half hours with her screaming and her tantrumming, and all she needed was a cuddle to go back down to sleep again. Whereas [family nurse] said, ‘no, just leave her to cry it out’. Whereas that might work with any other child, they’ll cry themselves to sleep.

While most parents who talked about ideas around infant crying saw these as important and/or relevant, there was one exception: a mother with primary school–aged children, who of all the respondents was most critical about parenting programmes (Interview 9). She argued that, in the interests of self-care, immediate attention to a crying infant was not always achievable:

R: So lack of stimulus and then the brain kind of just switches off and goes right, well, we won’t bother doing that any more. And that’s horrible.

I: Yes, I can imagine that’s quite depressing.

R: It’s really depressing, and then that led onto the picture of these poor children in the Romanian orphanage which was.…But again, another example of something that was just looking back. Fine, we all ignored our children for a little bit when they’ve been crying because actually, frankly, we just have to have five minutes peace. But there’s nothing I can do about it now.

Through the interview material presented above, we have shown that leaving a sleepy child to cry was commonly, though not exclusively, presented by our respondents as having neurologically and/or (psycho-)developmentally adverse consequences. Such presentations highlight the somatic significance of deliberate acts of care, similar to the tales of neglected Romanian orphans. We have evidenced how guidance relating to crying children was accounted for by our participants as changing over time, including during their own parental biographies, and how parents negotiated at times conflicting ideas. Such negotiations included forms of narrative work through which caregivers (re-)evaluated past and present parenting practices. Expert ideas and knowledge were adopted, adapted, and rejected based on both their do-ability (Fujimura, 1987) and their perceived applicability, underscoring again how everyday epistemologies of parenting, including understandings of children’s personalities and agency, shape engagements with other forms of knowledge.

## Discussion

Drawing on 22 interviews with parents/carers of (mostly) young children, we have examined how attendees of Scottish parenting programmes engage with ideas about infant neuropsychological development presented within these programmes. Significant to many of our respondents was the concept of processing speed, and specifically the notion that children’s brains process information seven times more slowly than those of adults (and hence take longer to comprehend requests). Several parents/carers also discussed hearing about Romanian infants who had been neglected in state orphanages in the 1980s, and reported that these infants had consequently experienced considerable (neuro)biological ramifications. Relatedly, respondents mentioned receiving advice about whether or not to leave their baby to cry to sleep, with parents usually noting that they had been encouraged to attend swiftly to their child since the infant might experience adverse neuroendocrinological effects. Understandings of childhood neurobiological limits and the somatic significance of love were evident across many of the interviews, with children’s subjectivities constituted, in part, through the functioning of their brain. Accounts of concepts like processing speed commonly presented childhood agency as having biological limits. Moreover, neglected infants were constructed as somatically different to those who had received parental love as expressed through carefully modulated voice, touch, and gaze.

It was not only respondents who explicitly mentioned brain development or the Romanian orphans that spoke about the need to interact with children. Often reflecting the content of the parenting programmes they had attended, in every interview participants discussed things like ‘being there’ for their children, and verbally and physically interacting with them. Examples of such interactive care included taking a child to the kitchen so they could watch their parents cook, rather than ‘plonking’ them in front of a television. This has resonance with Wall’s (2004, 2010) findings of US parents and carers who seek to use every minute with their child ‘productively’. However, the anxiety evident in the accounts of Wall’s interviewees was rarely explicit within the talk of our respondents.

Within the reflections of those interviewees who did mention brain development, neurologic subjectification was accounted for mostly as having positive consequences for caregivers and their children; that is, it activated affects like empathy. For instance, information about processing speed was described by some participants as encouraging them to be more patient with their children. Narratives conveying the biological benefits of attentive care (such as stories about the Romanian orphans or soothing one’s child rather than leaving it to cry) were sometimes also advocated as prompts to interact more with one’s children. Accordingly, many of our respondents experienced the brain-related information encountered within parenting programmes as affectively generative (see Beaulieu, 2003), expanding empathy and impelling renewed attention to care.

The facticity of the neuropsychological narratives presented to caregivers through parenting programmes was rarely explicitly criticised; however, their relevance to the respondents’ personal lives was subject to consideration and adjudication. In particular, neurologic notions were related only partially to the participants’ *own* children, as opposed to those of others. Moreover, participants’ talk also largely constructed children as having autonomy in spite of some biological limits to agency. For instance, notwithstanding children’s apparently slower processing speed, the respondents in one interview noted that their offspring could and did deliberately ignore them – they were not simply or solely processing parental commands more slowly. Accordingly, understandings based on neuropsychological knowledge could be reflexively rejected if they failed to resonate with everyday epistemologies of parenting (see Romagnoli and Wall, 2012). Indeed, some of the participants asserted that they received too much ‘advice’ from a range of actors in their networks, usually unsolicited, and noted that these different recommendations not uncommonly contradicted each other (reflecting, for instance, the different contexts in which advice/research is produced). For example, some parent respondents suggested that studies conducted in ‘extreme’ situations beyond the UK were not relevant for their local parenting practices.

In addition to geographical variation, parents, through the parenting programmes in which they had participated, engaged with science produced in different periods, such as evidence produced in the 1990s, attachment-based parenting that draws on (but also takes out of context, as Ramaekers and Suissa [2012] argue) the work of Bowlby (1969), and slightly newer developmental studies on processing speed (Kail, 2000). Parents did not usually construct the relevance of studies as a function of when they were conducted, although on occasion they did reflect critically on what one mother termed ‘out of date’ research, construing this as carrying less salience for contemporary parenting.

When negotiating neuropsychological notions, and discussing care more broadly, our participants also conveyed their understandings of what it meant to be a good parent. Generally, this was not someone who necessarily sacrificed themselves completely for their child, nor someone who focused all parental efforts exclusively on the optimisation of infant neurological development. Rather, a good parent was someone who strove to make ongoing physical and emotional contact with their children, in order to enhance current and future well-being (part of which related to neuropsychology). Such a characterisation is proximate to long-standing conceptions of (good) parents within British civil society (Duschinsky, Greco, and Solomon, 2015a, 2015b; Ramaekers and Suissa, 2012; Rose, 1998). In this respect, despite the biologised infant subjectivities we have documented in our respondents’ accounts, the neuropsychological knowledge operationalised within parenting programmes might best be regarded as intensifying and subtly shaping pre-existing tropes about how (best) to raise children, rather than as (necessarily) radically reconfiguring caregiving. What is clear, however, is that neither fulsome celebrations nor outright criticism of programmes that offer parents resources for the neuropsychological subjectification of children – their own or, especially, others’ – will adequately engage the diverse meanings participants invest in the knowledge and narratives they encounter therein.

## Conclusion

By drawing on contemporary Scottish interview data, this article is intended to contribute to long-standing debates on how humans think about themselves, how they incorporate scientific knowledge into such understandings, and how they may also recast and resist this knowledge as well. Child-rearing, which historically has been one of the dominant topics on which people seek out (popular) scientific knowledge (Mandler, 2019; Mechling, 1975; Ramaekers and Suissa, 2012), is an important case study for studying engagements with (neuro)science, and the extent to which, and ways in which, these engagements produce novel subjectivities. This is an ongoing debate in, and for, the history of the human sciences, partly indebted to Foucault’s work on power, knowledge, and subjectivity (Duschinsky, Greco, and Solomon, 2015a, 2015b; Harrington, 2016). Critical scholars have argued that neuroscientific knowledge has more potential to shape subjectivities because of its allure, even though enjoinders to parents that interpolate neurological ideas have not necessarily changed so much compared to previous decades of parenting advice. Our analysis extends other empirical and conceptual scholarship that describes the multiplicity of epistemologies and ontologies that form contemporary subjectivities, within which the neurosciences are not, as a matter of course, dominant but rather co-exist with and further complicate other ways of understanding what it means to be human (Gardner *et al.*, 2018; Pickersgill, Cunningham-Burley, and Martin, 2011; Pickersgill, Martin, and Cunningham-Burley, 2015; Singh, 2013; Smith, 2019).

## Note

1. Or rather, mothers, since most of these studies only ever investigate mothers’ attitudes – thereby implicitly reinforcing an understanding of female parents as being ‘in charge’.
